# An ambulance for retinol

**DOI:** 10.7554/eLife.04246

**Published:** 2014-09-02

**Authors:** Stephanie C Ganal, Andrew J MacPherson

**Affiliations:** 1 **Stephanie C Ganal** is in the DKF Maurice Müller Laboratories, University of Bern, Bern, Switzerland; 2 **Andrew J MacPherson** is in the DKF Maurice Müller Laboratories, University of Bern, Bern, Switzerlandandrew.macpherson@dkf.unibe.ch

**Keywords:** retinol transport, serum amyloid A, acute phase response, crystal structure, *E. coli*, mouse

## Abstract

During inflammation, serum amyloid A proteins transport retinol to infected tissues.

**Related research article** Derebe MG, Zlatkov CM, Gattu S, Ruhn KA, Vaishnava S, Diehl GE, MacMillan JB, Williams NS, Hooper LV. 2014. Serum amyloid A is a retinol binding protein that transports retinol during bacterial infection. *eLife* **3**:e03206. doi: 10.7554/eLife.03206

**Image** When mice are fed a diet rich in vitamin A, serum amyloid A proteins (red) accumulate in the cells lining the small intestine

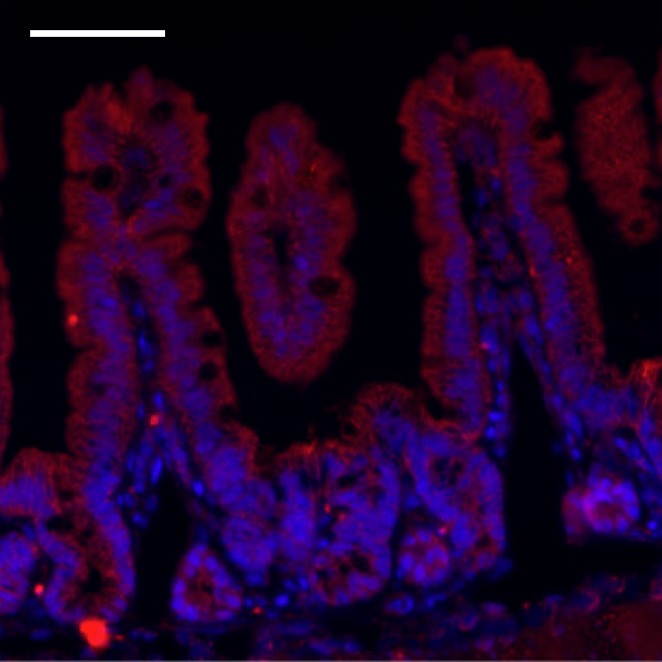

The serum amyloid A (SAA) proteins produced in response to inflammation are well known for the damage that they can cause during diseases. Chronic infections and autoimmunity or auto-inflammatory disorders (such as familial Mediterranean fever) can result in a condition called amyloidosis, where long fibres of SAA are deposited in the liver, kidney, intestine and nervous system. Such amyloid deposition causes serious damage to these organs, which exacerbates the original disease (Eklund et al., 2012).

With such serious potential consequences, there must be excellent reasons for keeping SAA proteins in the genome, but these reasons were unclear. Now, in *eLife,* Lora Hooper and colleagues at the University of Texas Southwestern Medical Center and the New York University School of Medicine report that SAA proteins play an important role in transporting retinoids during an infection (Derebe et al., 2014).

Our diet needs to contain naturally occurring plant pigments called carotenoids, which include the molecules that can form vitamin A. Strictly speaking, vitamin A is a molecule called retinol, although it can be converted through oxidation into retinal and retinoic acid, which are also biologically active. These retinoids are involved in many developmental and physiological processes, such as bone growth and development, reproduction, normal vision and the immune response against infections (D'Ambrosio et al., 2011). Childhood death from measles, and from respiratory or intestinal infections, is a common problem in areas of widespread vitamin A deficiency (Sommer, 2008).

The body takes up dietary retinoids (including retinyl esters, retinol and proretinoid carotenoids) through the absorptive cells that line the upper small intestine. Retinyl esters and carotenoids are converted to retinol either before or during entry to these cells (Figure 1). Retinol can then be transported around the body by binding to a serum retinol binding protein, and is stored mainly in the liver. From here, it is recruited to other tissues upon demand (D'Ambrosio et al., 2011).

**Figure 1.**
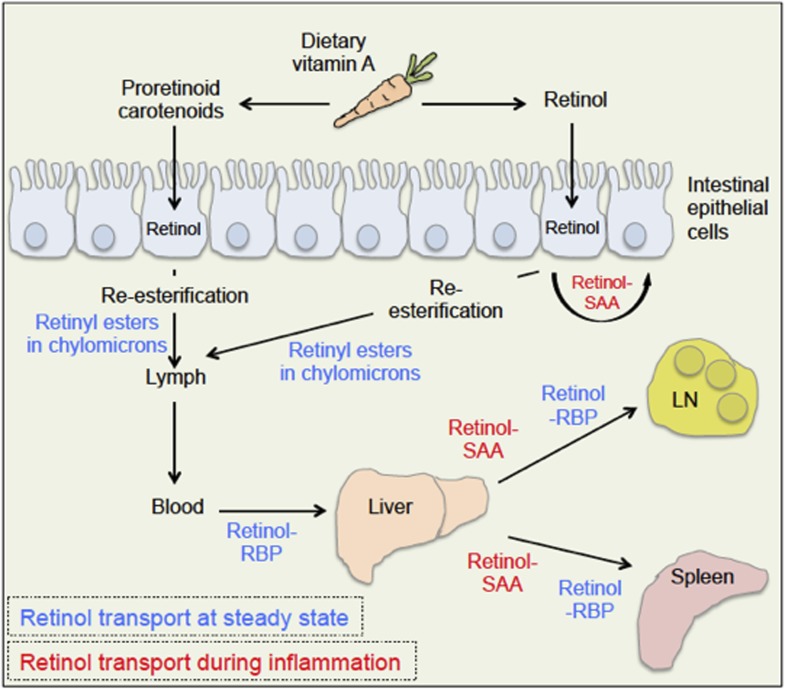
Retinoid transport is different in health (blue) and disease (red). Carrots contain molecules that are converted into retinol (‘vitamin A’) in the upper small intestine. Retinyl esters are converted to retinol either inside the intestine or at the intestinal epithelial cell ‘brush border’ on the surface of the cells. Carotenoids are ultimately reduced to retinol after entering the absorptive cells lining the intestine. When an animal is healthy, retinol molecules in the intestinal epithelial cells go through a process called re-esterification, and are packed into particles called chylomicrons. These move out of the epithelial cells and into the lymph and blood. Retinol then binds to a serum retinol binding protein (RBP), which moves retinol to the liver, where retinol is stored until it is needed. To leave the liver, retinol again binds to a retinol binding protein, and may be transported to a range of locations, including the lymph node (LN) and the spleen. During inflammation, levels of the retinol binding protein drop; and Derebe, Zlatkov et al. have found that retinol is instead transported out of the liver to where it is needed by serum amyloid A (SAA) proteins.

A time when retinol is in great demand is during an infection. Large amounts of retinol are required in the lymphatic tissues—which contain the white blood cells that defend the body against microbes—in order to mount an efficient immune response (Hall et al., 2011b). Paradoxically, levels of serum retinol binding protein are strongly reduced during an infection (Rosales et al., 1996). Therefore, how large amounts of retinol are delivered to infected target tissues was an unresolved question. Hooper and co-workers—including Mehabaw Derebe and Clare Zlatkov as joint first authors—have now elegantly shown that the SAA proteins are potent retinol binding proteins that transport retinol to help the body battle microbial infections (Derebe et al., 2014).

SAA proteins are found in blood plasma, and are produced mainly by the liver and intestine. Four different genes for SAA proteins are present in the genome of mice and humans, although one of these—*SAA3*—no longer seems to produce proteins in humans. SAA-deficiency has never been described, suggesting that the SAA proteins are critical for survival (Eklund et al., 2012).

The production of two types of SAA protein—SAA1 and SAA2—is massively increased in the liver during the immediate immune response following an infection or during microbial colonization of the intestinal epithelium (Morrow et al., 1981; Ivanov et al., 2009; Reigstad et al., 2009; Derebe et al., 2014). SAA proteins can bind to and activate several cell surface receptors on myeloid cells, which are the first cells to be activated during an immune response. The receptors produce several different signalling molecules involved in stimulating various parts of the immune response. SAA proteins thereby efficiently contribute to combating inflammation (Eklund et al., 2012).

Derebe, Zlatkov et al. compared the genes expressed in the intestinal epithelial cells of mice fed a diet either containing or lacking vitamin A. This revealed changes to the RNA levels expressed by a series of genes; the genes that produce the SAA proteins were amongst those affected. The expression of SAA proteins in the intestine and the liver was also strongly enhanced by the amount of vitamin A the mice consumed. Although the sequence of amino acids that makes up SAA is not similar to the sequences found in other known retinol binding proteins, it does contain a predicted retinol-binding surface. From this and the fact that SAA proteins are induced in the presence of retinol, Derebe, Zlatkov et al. suspected that they could be dealing with retinol-binding proteins.

The hypothesis that SAA proteins are retinol-binding proteins was neatly confirmed in several ways. Both human SAA1 and mouse SAA1 and SAA3 were seen to bind retinol tightly *in vitro*. Additionally, retinol was found bound to SAA extracted from the serum of mice infected with a species of Salmonella bacteria.

Derebe, Zlatkov et al. then solved the crystal structure of mouse SAA3. This consists of four α-helices in a cone shape, and is very similar to the recently described structure of human SAA1 (Lu et al., 2014). Four SAA3 molecules bind together in solution to form a hydrophobic central channel that, according to predictive binding studies, is able to bind retinol. Derebe, Zlatkov et al. were able to underline their prediction by changing one of the amino acids in the hydrophobic core of mouse SAA3; this reduced its ability to bind to retinol.

These results give us a new insight into the probable mechanism behind the roles of both vitamin A and SAA proteins in combating infection (Hall et al., 2011b). We know that SAAs are required to generate a type of immune cell called Th17 that targets microbes in the gut. In addition, mice deficient in *Saa1* or *Saa2* are more susceptible to chemically-induced colitis (Eckhardt et al., 2010). Derebe, Zlatkov et al. have now also shown that *Saa1/2*-deficient mice infected with Salmonella harbour fewer bacteria in the spleen and liver than wild-type controls. Previously, SAAs were only known to be involved in the immune response in mucous membranes: this extends the known functions of SAAs to systemic immune responses as well.

Future studies will clarify which of the currently known functions of SAA proteins depend on their capacity to transport retinol. For example, SAA proteins were reported to play a role in the generation of Th17 cells; however, retinol and retinoic acid themselves are crucial for generating Th17 cells during infection and mucosal challenges (Ivanov et al., 2009; Hall et al., 2011a). So is this function of SAA proteins solely due to the fact that they deliver retinol to the lymphoid tissue? In addition, do different SAA proteins have distinctive roles: for example, do they transport retinol to different specific target tissues?

Overall, the findings of Derebe, Zlatkov et al*.* nicely solve one aspect of the highly important retinoid pathway in immunity: namely, how retinol is transported during a microbial challenge to meet the increased demand for signalling in lymphoid tissues during an immune response.
